# Spontaneous Assembly of an Organic–Inorganic Nucleic Acid Z‐DNA Double‐Helix Structure

**DOI:** 10.1002/anie.201606658

**Published:** 2016-11-30

**Authors:** Vladislav Kulikov, Naomi A. B. Johnson, Andrew J. Surman, Marie Hutin, Sharon M. Kelly, Mohammed Hezwani, De‐Liang Long, Gerd Meyer, Leroy Cronin

**Affiliations:** ^1^ The University of Glasgow WestChem School of Chemistry Joseph Black Building Glasgow G12 8QQ UK; ^2^ Universität zu Köln Institut für Anorganische Chemie Greinstrasse 6 50939 Köln Germany; ^3^ Iowa State University Department of Chemistry Ames Iowa 50011 USA; ^4^ The University of Glasgow Institute of Molecular Cell and Systems Biology Joseph Black Building Glasgow G12 8QQ UK

**Keywords:** evolution of evolution, helical structures, nucleobases, polyoxometalate hybrids, self-assembly

## Abstract

Herein, we report a hybrid polyoxometalate organic–inorganic compound, Na_2_[(HGMP)_2_Mo_5_O_15_]⋅7 H_2_O (**1**; where GMP=guanosine monophosphate), which spontaneously assembles into a structure with dimensions that are strikingly similar to those of the naturally occurring left‐handed Z‐form of DNA. The helical parameters in the crystal structure of the new compound, such as rise per turn and helical twist per dimer, are nearly identical to this DNA conformation, allowing a close comparison of the two structures. Solution circular dichroism studies show that compound **1** also forms extended secondary structures in solution. Gel electrophoresis studies demonstrate the formation of non‐covalent adducts with natural plasmids. Thus we show a route by which simple hybrid inorganic–organic monomers, such as compound **1**, can spontaneously assemble into a double helix without the need for a covalently connected linear sequence of nucleic acid base pairs.

The central dogma of molecular biology is built upon the DNA duplex.^1^ Perfectly aligned so that its two linear information polymer strands can be non‐covalently joined to their mutual complements, the unwinding of the two DNA strands and their subsequent replication provides the mechanism for the perpetuation of genetic information in biology.^2^ However, as elegant and profoundly simple as this system is, the means by which the first DNA duplexes formed remain unclear.^3^ One possibility could be that self‐replicating minimal “inorganic” materials were able to spontaneously form a system capable of bridging this gap, leading to the emergence of living systems.^4^ Thus the very basic evolutionary information could have been encoded in naturally occurring periodic systems in the form of the directionality of crystalline layers, lattice defects, or chirality, for example. This “information” could then be transferred to molecules adsorbed on the surface of the material but this theory has lacked evidence or an experimental framework for development since it was proposed by Cairns‐Smith in 1966.^4^ We hypothesized that the formation of simple hybrid inorganic–organic units provides a model of an intermediate class that electrostatically assemble into structures possessing characteristics similar to those of the information copying motifs found in biology.^5^

To explore this idea, we set about synthesizing a very simple prototype nucleobase–metal oxide hybrid: guanosine monophosphate with a {Mo_5_O_15_}‐based polyoxometalate. This compound, Na_2_[(HGMP)_2_Mo_5_O_15_]⋅7 H_2_O (**1**), was formed by the condensation reaction of guanosine monophosphate (GMP, **2**) and sodium molybdate upon acidification.^6^ To our surprise, we found that compound **1** reproducibly forms an exceptionally intricate crystalline structure with similar dimensions to those of Z‐DNA, but without hydrogen‐bond base pairing (see Figure 1 and Table 1; for a comprehensive version, see the Supporting Information, Table S2). The compound crystallizes in the space group *P*6_5_22 containing a sixfold screw axis with a left‐handed twist and two orthogonal twofold rotational axes as symmetry elements. Within the structure, the anions consisting of an inorganic POM core and two ligands protruding from opposing sides are situated along the resulting helix, and are interconnected by a row of Na^+^ cations.

**Figure 1:**
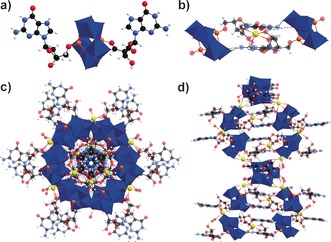
a) The hybrid anion of **1**. b) Interconnection of two hybrid anions by hydrogen bonding, stacking interactions, and Na^+^ coordination, forming a dimer. c, d) One helical turn of **1** as viewed along the crystallographic *c* and *b* axes. C black, H white, N light blue, O red, P orange, Mo blue polyhedra, Na yellow.

**Table 1:** Structural features of ideal B‐ and Z‐DNA as well as **1**.

	B‐DNA^10^	Z‐DNA^10^	Compound **1**
helical sense	right‐handed	left‐handed	left‐handed
diameter	ca. 20 Å	ca. 18 Å	ca. 32 Å
BP^[a]^ per turn	10 pairs	6 pairs	6 pairs^[b]^
twist per BP^[a]^	36°	60° per dimer	60° per dimer
rise per turn	34 Å	45 Å	42 Å
rise per BP^[a]^	3.4 Å	7.4 Å per dimer	7.0 Å per dimer
interplanar ring distance	3.4 Å	3.4 Å within dimers	3.4 Å within dimers
sugar pucker	C2′‐*endo*	C2′‐*endo* for pyrimidines; C3′‐*endo* for purines	C2′‐*endo*
glycosidic bond	*anti*	*anti* for pyrimidines; *syn* for purines	*anti*

[a] BP=base pair. [b] Pairs of guanine bases not participating in classical base pairing.

The inorganic moiety consists of a ring of five condensed molybdate(VI) anions capped by phosphate groups above and below the ring plane in the so‐called Strandberg geometry.^7^ The guanosine is connected to this moiety via the oxygen atom on the ribose ring at position 5′ (Scheme S1). The organic ligands are connected to the neighboring polyoxoanions through hydrogen bonds donated by the amino group, forming a dimer (Figure 1 b). Stacking interactions between the guanine rings and chelate coordination of Na^+^ cations by the hydroxy groups of the ribose ring reinforce the linkage. The sixfold symmetry axis runs through the center of the dimers, creating a left‐handed double helix (Figure 1 c, d and Movie S1).

The different major conformations of DNA, namely A‐, B‐, and Z‐DNA (Figure S1), can be differentiated by their geometrical parameters (see Table 1 and Table S2).^8^ The symmetry and geometrical parameters of **1** are closely related to the Z‐form of DNA. The tendency of a DNA sequence to form the Z‐conformation increases with abundance of guanosine.^9^ Further, the rise per turn and incline towards the central symmetry axis are very similar in these structures. Both compound **1** and Z‐DNA form dimers that wind around the sixfold symmetry axis with left‐handed helicity; however, these are different in their nature.^10^ The dimers of the guanosine Strandberg anion result from direct hydrogen bonding between the ligand and the POM (Figure 1 b), whereas dimers of Z‐DNA consist of two purine–pyrimidine base pairs, where only the organic moieties are hydrogen‐bonded. Furthermore, in **1**, the guanosine ligands are stacked in an antiparallel orientation. The geometry of the ribose rings in **1** and Z‐DNA determines the overall shape of the macromolecule and thus plays a decisive role in the refinement of the data obtained for different forms of DNA by fiber X‐ray diffraction.8a, 11 The C2′‐*endo* conformation is assumed by the ribose rings of **1**, as coordination of a Na^+^ ion by two oxygen atoms is only possible in this conformation. It is important to note that the Z‐form of DNA is stabilized by high salt concentrations as cations effectively shield the negative charges of opposing strands from one another (i.e., the phosphate–phosphate distance decreases from 12 Å in B‐DNA to 8 Å in Z‐DNA),^2^ and a similar effect can be observed in the structure of **1**.

The exceptional aspect of structure **1** is the close resemblance to the iconic helical DNA structure. In previous work involving helical structures, they were also compared with that of DNA:^12^ this includes studies that show the ability of nucleotide‐like organic molecules to arrange themselves into DNA double‐helix dimensions without the need for a backbone.^13^ However, none of the previously reported organic–inorganic structures display helical parameters that approach those of any of the major DNA conformations. Compound **1**, on the other hand, can be overlaid in a helical sense as the number of dimers per turn, helical pitch, and rise per turn all map almost perfectly onto the Z‐form of DNA, marking structure **1** as particularly unusual (see Table 1 and Table S2).^14^

Upon a more detailed structural analysis, we found that of the two major components of the hybrid anion, only guanosine displays chiral centers and hence the ability of building homochiral structures. The “simple” compounds like hydrated Na_2_GMP (**3**) and H_2_GMP (**4**) crystallize in the chiral space group *P*2_1_2_1_2_1_, but do not form helical structures like **1**.^15^ The guanine rings are hydrogen‐bonded to the ribose or water molecules instead. According to a CCDC search, there are 46 monomeric structures of GMP and derivatives thereof, 24 of which are transition‐metal complexes of GMP. Some of them crystallize with polar or chiral symmetries, but no base pairing with stacking interactions has been observed. As such, compound **1** is the first non‐oligomeric GMP derivative crystallizing with sixfold symmetry to form a double helix similar to the DNA duplex, and the helical parameters are almost identical to those of Z‐DNA. In contrast, many other DNA models with or without nucleotides display different helicity, symmetry, and parameters, such as rise and number of dimers per turn, or have no DNA backbone substitute.12a, 16 It is worth noting that the adenosine analogue of **1**,6b Na_2_[(HAMP)_2_Mo_5_O_15_]⋅6 H_2_O (AMP=adenosine monophosphate), which crystallizes in the space group *P*3_1_21 with a threefold twist axis going through the middle of the adenine rings, does not bear any structural resemblance to Z‐DNA.

The most intriguing question posed by the above structure is whether compound **1** remains intact in aqueous solution, and whether the helical structure is preserved. To investigate this, we employed several analytical techniques to probe the nature of compound **1** in solution (maintained acidic as hydrolysis is observed at neutral pH). As polyoxometalate anions can easily speciate in aqueous solution, we explored the solution stability of the hybrid anions in water by ^31^P NMR spectroscopy (Figure S5). The ^31^P resonance appears as a multiplet in the NMR spectrum, confirming the persistence of the P−O−R bonds of **1** in water. Furthermore, IMS‐MS analysis revealed a series of oligomeric peaks, which may be assigned to a [(GMP)_2_(Mo_5_O_15_)_1_(K)_*W*_(Na)_*X*_(H)_*Y*_(H_2_O)_*Z*_]_*m*_ series (GMP=C_10_H_13_N_5_O_8_P), further corroborating the persistence of **1** in solution and its tendency to self‐associate (see Figure S7).

To examine the conformation of the extended structure of **1** in solution, we employed circular dichroism, a technique that is widely used to investigate the structure of DNA and other biomolecule assemblies in solution (Figure 2). At room temperature, only a weak signal is observed; however, upon cooling, a distinctive pattern emerges with maxima around 210 and 260 nm, showing the formation of a more structured framework as the solution becomes less dynamic. Upon reheating, the original structure does not reform. Instead, the character of the 5 °C spectrum remains, where the intensities of the differential absorption bands are reduced but their pattern remains unchanged. This is indicative of a well‐defined secondary structure. However, this pattern does not include features consistent with the structure of Z‐DNA (negative minimum at 290 nm and positive maximum at 260 nm). This is perhaps unsurprising as the structural similarity of **1** and Z‐DNA results from different interactions, and neither confirms nor negates the persistence of the helical structural motif in solution.^17^ As **1** is a derivative of guanosine monophosphate (**2**), we compared its CD spectrum to that of **2** (also measured in an acidic medium), which showed that they are clearly different. For example, at 210 nm, a maximum is observed in the spectrum of **1** while a minimum is observed for **2**, and a maximum is observed around 260 nm for **1**, whereas no such feature is observed for **2**.^18^ Inferences from CD on the secondary structure of **1** proved elusive, perhaps as the acidic pH value prevents many of the hydrogen‐bonding interactions that define more well‐known solution structures.

**Figure 2:**
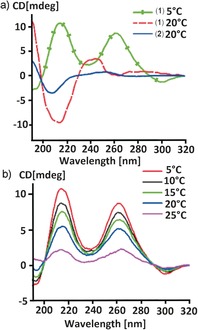
a) CD spectra of POM‐GMP (**1**) in a 0.01 cm cell at 20 °C and a 0.001 cm cell at 5 °C and the GMP (**2**) reference spectrum in a cell with a path length of 0.01 cm (blue). b) Temperature dependence of the CD spectrum of **1** after cooling to 5 °C. All spectra were recorded at a concentration of 4 mm and pH 1.2. See Figure S6 for the respective HT [V] spectra.

AFM data was obtained after drop‐casting a solution of **1** on a freshly cleaved mica surface (see the Supporting Information for the exact conditions). The resulting fibers display a noticeable helical twist (Figure 3 a). However, the features seen on the surface by AFM depend strongly on the local concentration. In some areas, a tight and systematic network of fibers with a height of around 3.5 nm is observed (Figure S11). Guanosine self‐assembly has already been studied by AFM and is known to result in fiber‐like structures on mica.^19^ In most reports, the height of these fibers is between 1.5–2.0 nm. The height of 3.5 nm measured in this study is a consequence of the inorganic core, and is consistent with the helicoidal diameter measured in the crystal structure (3.20 nm). It is expected that the interactions between **1** and the surface are mediated by Na^+^ cations as freshly cleaved mica surfaces are negatively charged.

**Figure 3:**
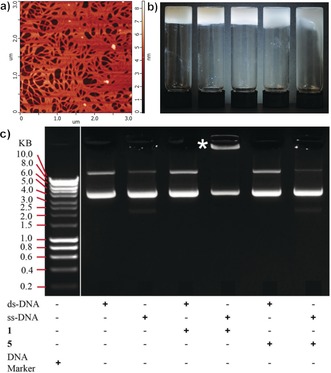
a) AFM image of the agglomerates of **1** on mica surface, taken in semi‐contact mode under air. b) Tube inversion test (pH 1.2). Concentrations of **1** from left to right: 0.025, 0.018, 0.014, 0.011, and 0.009 mol L^−1^. c) DNA interactions with **1**. ds‐DNA=double‐stranded pGLO plasmid DNA, ss‐DNA=single‐stranded pGLO plasmid DNA. The new band appearing upon interaction of the ss‐DNA with **1** is marked with *. See the Supporting Information for the exact conditions.

The ability of **2** to act as a low‐molecular‐weight gelator (LMWG) is well documented and due to its tendency to build macromolecular aggregates.^20^ A more pronounced version of this behavior might be expected for **1** as other Strandberg‐type inorganic–organic hybrids have also shown gelator properties.^21^ To test this, we chose the most pragmatic test to characterize the LMWG properties of **1**—the tube inversion method (Figure 3 b). The critical gelation concentration (CGC) of **1** at room temperature is 0.009 m at pH 1.2 (the right‐most vial in Figure 3 b). This value corresponds to 1.28 wt %, which is striking given the high molecular weight of **1** compared to other LMWGs.^22^ Other POM hybrids have also been shown to act as gelators,^21^ but none formed hydrogels. Given the fact that pure **2** does not form hydrogels even at 0.3 m (50 times the CGC of **1** at pH 1.2) at neutral pH, one could assume that synergistic supramolecular interactions between the organic and inorganic moieties of **1** are responsible for gel formation.

The interactions between polyoxoanions and biomolecules are not only of general interest, but could also be useful for the development of POM‐based drugs and chemical biology tools.^23^ To evaluate the ability of **1** to interact with a functional biopolymer, the hybrid compound was incubated with double‐stranded (ds) and single‐stranded (ss) plasmid DNA (pGLO). No effect on the DNA migration during electrophoresis was detected for ds‐DNA, but a new band was observed for ss‐DNA (Figure 3 c, lanes C and D). No interaction with either ss or ds plasmid DNA was detected after incubation of the inorganic Strandberg anion Na_6_Mo_5_P_2_O_23_ (**5**; Figure 3 c, lanes E and F).^7^ The presence of the new band after incubation of the ss‐DNA with **1** and its absence following the incubation with the inorganic core anion **5** suggests an interaction between the free guanosine ligands on **1** and single‐stranded DNA.

In conclusion, the most intriguing feature of compound **1** is its structural similarity to Z‐DNA.^17^ We have shown that neither 1) a covalent backbone nor 2) hydrogen‐bond‐mediated base pairing is necessary for the build‐up of homochiral helical structures, and that 3) guanosine seems to strongly influence the rotational direction of the helix and the overall structural geometry. This is interesting as it has often been said that at some point in evolutionary history, an information‐carrying biopolymer “must have arisen based on purely chemical means”.^25^ Herein, we have described a new class of compounds that spontaneously forms an analogue of a nucleic acid double helix. This takes place without the need for a pre‐programmed or biochemically written linear sequence of nucleic acid base pairs. The similarities of the extended structure of **1** and Z‐DNA are unprecedented, with no other simple nucleobase monomer or hybrid showing such a structure out of over 750 000 entries in the Cambridge Structural Database. Furthermore, this work shows that biochemical machinery is not required to produce a double helix with this degree of similarity to Z‐DNA. Given the current gap between the inorganic and biological world, we would like to suggest that hybrids such as **1** might offer a bridge between the inorganic and biological worlds as depicted in Figure 4. For instance, Benner has shown that the formation of ribose is promoted by molybdate under acidic conditions similar to those required to form **1**.^26^ We postulate that the key to understanding the “evolution of evolution” is exploring avenues that reduce the information required such that a spontaneously assembled double helix can become programmable, that is, become an evolvable polymer sequence. This is currently under investigation in our laboratory along with the search for the simplest synthetic conditions that yield the monomers.

**Figure 4:**
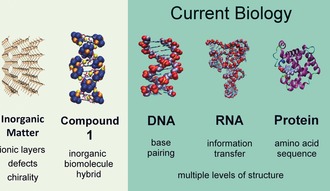
The increase in complexity from inorganic materials to DNA, RNA, and proteins (from left to right). We therefore suggest that the inorganic–organic hybrid **1** can be seen as an example system that can be placed between the spontaneously forming “inorganic” world and the evolvable “biological” world.^24^

*In memory of A. Graham Cairns‐Smith (1931–2016)*

## Supporting information

As a service to our authors and readers, this journal provides supporting information supplied by the authors. Such materials are peer reviewed and may be re‐organized for online delivery, but are not copy‐edited or typeset. Technical support issues arising from supporting information (other than missing files) should be addressed to the authors.

Supplementary

Supplementary

Supplementary
