# Supplementary material for: Spontaneous Assembly of an Organic–Inorganic Nucleic Acid Z‐DNA Double‐Helix Structure
Source: Angew Chem Int Ed Engl. 2016 Nov 30;56(4):1141–5. doi: 10.1002/anie.201606658 (PMC6057607; doi:10.1002/anie.201606658)
Supplement: Supplementary file 1 — Supplementary [file ANIE-56-1141-s001.pdf]

Angewandte Chemie
Eine Zeitschrift der Gesellschaft Deutscher Chemiker

Supporting Information

Spontaneous Assembly of an Organic–Inorganic Nucleic Acid Z-DNA Double-Helix Structure

*Vladislav Kulikov, Naomi A. B. Johnson, Andrew J. Surman, Marie Hutin, Sharon M. Kelly, Mohammed Hezwani, De-Liang Long, Gerd Meyer, and Leroy Cronin**

anie_201606658_sm_miscellaneous_information.pdf
anie_201606658_sm_Movie_S1.mov
anie_201606658_sm_Movie_S2.mov

Table of Contents

1. General Experimental Section 2
2. Synthesis of 1 4
3. X-ray Crystallography 4
4. Supporting Illustrations 5
5. NMR Spectroscopy 9
    5.1 NMR Signals 9
    5.2 P^{31}-NMR Spectrum 9
    5.3 Low field region of the ^{1}H-NMR Spectrum 10
6. Ion mobility - Mass Spec 11
7. Circular and Linear Dichroism 11
8. Atomic Force Microscopy 12
    8.1 Three days solution ageing 12
    8.2 Five days solution ageing 13
9. DNA Annealing Experiments 15
10. Differential Thermal Analysis / Thermogravimetry 16
11. Gel Electrophoresis 16
12. List of Movies 17
13. References 17

1. General Experimental Section

The starting materials for the synthesis of **1** were purchased from commercial sources and used without further purification. Compound **1** was previously obtained by Young *et al.* in a similar reaction procedure, but only characterized in the powder form and in solution.[1]

NMR-Spectroscopy: The NMR-spectra were measured on a Bruker 400MHz spectrometer. The samples were prepared from D_2O/H_2O (1/10) solutions, the pD/pH adjusted by DCl/D_2O (0.2 M). The NMR spectra are referenced to the peaks of 3-(Trimethylsilyl)propionic-2,2,3,3-d_4 acid (87 mM, δ (H, C of the TMS-group) = 0.0 ppm) and D_3PO_4 (1 mM, δ = 0.0 ppm) in D_2O. The assignment of ^{1}H and ^{13}C NMR signals was verified by ^{1}H,^{1}H-COSY and ^{13}C,^{1}H-HSQC measurements.

CD-Spectroscopy: CD Spectra were recorded in a JASCO J-810 spectropolarimeter in quartz cells using pathlengths ranging from of 0.001 cm to 0.01cm.

AFM: The AFM pictures were taken in semi-contact mode using the NTEGRA Spectra platform of NTMDT. The cantilevers used were purchased from NTMDT (NSG10, resonant frequency 140-390 kHz, force constant 3.1-37.6 N/m). The samples were prepared by drop cast 10 µl of the solution of 1 (0.1 mg/mL, pH = 1.95 adjusted by 1 M HCl, matured for 6 days) on a freshly cleaved mica surface.

Elemental Analysis: Elemental analyses for carbon, hydrogen and nitrogen were performed on the Vario EL analysing machine (Elementaranalysesysteme GmbH).

ICP-OES: 30mg of **1** was submitted to the *Institut für Festkörperforschung* in Jülich, Germany, for analysis of Mo, Na and P. Sample was digested in a 3:1 mixture of HNO_3 and H_2O_2. A *TJA-IRIS-Advantage* spectrometer with echelle optics and CID semiconductor was used to observe in the wavelength range 170 – 900 nm.

Tube Inversion Tests: The samples for inversion test were prepared by addition of HCl (1M) to the solutions of Na_2MoO_4•$2H_2O$ (**6**) and Na_2GMP•H_2O (**3**) after which the purity of **1** was verified by ^{31}P NMR. Each sample was left standing for 20 min every time before conducting the inversion test.

Differential Thermoanalysis / Thermogravimetric Analysis: DTA/TG-measurements were performed on a Netzsch STA 409 thermal analyser with a heating rate of 10°C/min.

DNA-Annealing Experiments: The interaction of **1** and **5** with DNA was assessed by incubating of 9 µg of each compound with double stranded (ds) and single stranded (ss) plasmid DNA (pGLO, 500µg in each case; in 12.6 mM acetate buffer at pH = 4.0) and resolving the complex formation on a 1% agarose TAE gel adjusted to contain 0.5 µg/mL ethidium bromide.

Electrophoresis: The electrophoresis studies were performed using a commercially available submarine-type electrophoresis system (Pt-wire electrodes set at a distance of 13 cm).

Ion-Mobility Spectrometry - Mass Spectrometry: IMS-MS spectroscopy samples were prepared by dissolving pure crystals of **1** in HPLC grade water, at approx. 1 mg/ml; these solutions were filtered and analysed immediately with no further purification (n.b. similar spectra obtained on dissolving samples in a water/acetonitrile mix with 2% formic acid, without the requirement for immediate analysis to avopid decomposition). All spectra were acquired on a Waters Synapt G2 HDMS instrument, with samples infused into the standard ESI source at 5 µl/min using a Harvard syringe pump. The following parameters were used for acquisition of all spectra (unless otherwise stated): ESI capillary voltage, 2.7 kV; sample cone voltage, 30 V; extraction cone voltage, 4.0 V; source temperature, 80℃; desolvation temperature, 180℃; cone gas (N2) flow, 15 L/h; desolvation gas (N2) flow, 750 L/h; source gas flow, 0 mL/min; trap gas flow, 2 mL/min; helium cell gas flow, 180 mL/min; IMS gas flow, 90 mL/min; IMS wave velocity, 1000 m/s; IMS wave height, 40 V. Data was acquired using MassLynx v4.1 and initially visualised using DriftScope v2.2.

IMS-MS spectra were further processed using UniDec[19] to allow clear visualisation and produce mass distribution spectra (i.e. deconvoluted “neutral mass spectra”). Briefly, the data processing workflow ran as follows (i) raw data files were loaded into UniDec; (ii) some filtering/processing was carried out – primarily subtraction of a curved background and gaussian smoothing in the m/z domain and application of a 5-10% minimum intensity threshold; (iii) peak width and shape was assigned using the UniDec GUI’s dedicated tool; (iv) manual assignment of peak charge was made in

most cases, where charge was clearly observable; (v) deconvolution was run, yielding 'cube' figures of the IMS-MS data, and deconvoluted "neutral mass" spectrum.

2. Synthesis of 1

Na_2MoO_4•$2H_2O$ (**6**, 0.72 g, 3.04 mmol, Merck, Darmstadt, Germany, p.a.) and Na_2GMP•H_2O (**3**, 0.61 g, 1.44 mmol, ABCR, Karlsruhe, Germany, 98%) were dissolved in H_2O (10 ml). 1.0 M HCl (7.2 ml) was added upon vigorous stirring over the course of 15 min adjusting the pH to 3.24. H_2O (6 ml) was added after further 50 min of stirring to the reaction mixture. The stirring was continued for further 30 min. White solid was obtained upon MeOH-vapour diffusion after a week. The white product was separated from the gelatinous reaction mixture via centrifugation (1600 rpm, 1 hr.) and washed 3 times with 20 ml portions of MeOH. 0.70 g (0.43 mmol, 58%) of the product was obtained after drying it for two days in air and two hours under dynamic vacuum (0.02 mbar). **IR** (KBr): ν (cm^{-1}) = 3350 (very broad, vs, 2938 (w), 1696 (s), 1635 (s), 1599 (s), 1533 (m), 1480 (w), 1411 (w), 1361 (m), 1250 (w), 1139 (s), 1073 (s), 993 (s), 932 (s), 907 (s), 798 (w), 681 (broad, vs), 526 (w), 496 (w). **Elemental Analysis (%):** Calc. for $C_{20}H_{40}Mo_5N_{10}Na_2O_{38}P_2$: C 14.86, H 2.49, N 8.67, Mo 29.68, Na 2.84, P 3.83; found: C 15.05, H 2.31, N 8.77, Mo 28.8, Na 3.38, P 3.63.

3. X-ray Crystallography

The crystallographic data of **1** are available in Table S1. Single crystals suitable for SC X-ray diffraction studies were obtained from a similar reaction mixture upon addition of 1 eq. of $[N(n\text{-}Bu)_4]Br$ to the centrifugate and subsequent methanol diffusion. Suitable single crystals of **1** were attached to a thin glass fibber by using Fomblin YR-1800 oil and mounted on a goniometer head in a general position. All data were collected at 150 K on an Oxford Gemini A Ultra diffractometer, with graphite monochromated X-radiation ($Mo_{K\alpha}$, λ = 0.71073Å), running under the CrysAlisPro software. The corrections for the incident and diffracted beam absorption effects were applied using analytical methods.[2,3] Structures were solved using Direct methods with SHELXS-97[4] using WinGX routines.[5] Structure refinement was accomplished by full matrix least-squares on F^2 via SHELXL-2013.[6] Non-hydrogen atoms except O-atoms of water, O6G and Na^+ were refined anisotropically. Hydrogen atom positions were calculated using standard geometric criteria and refined on riding model. All data manipulations were performed using WinGX.[5] Crystallographic data have been

deposited with the Cambridge Crystallographic Data Centre, CCDC, 12 Union Road, Cambridge CB21EZ, UK. Copies of the data can be obtained free of charge on quoting the depository numbers CCDC 1029199 (Fax: +44-1223-336-033; -Mail: deposit@ccdc.cam.ac.uk, http://www.ccdc.cam.ac.uk).

Formula	$C_{20}H_{40}Mo_5N_{10}Na_2O_{38}P_2$	ρ_{calcd} [g•cm^{-3}]	1.686
M_r	1616.24	μ [mm^{-1}]	9.306
T [K]	150(2)	Refl. collected/unique	75586/4943
Crystal system	Hexagonal	R_{int}	0.0394
Space group	*P*6$_5$22	Refined parameters	357
a [Å]	16.0517(3)	Flack x-Parameter*	0.005(8)
c [Å]	42.8082(7)	GooF	1.043
V [Å^3]	9552.1(4)	R1(I>2σ(I))	0.0888
Z	6	wR2(all)	0.2637

Table S1. Crystal and structure refinement data for $Na_2[(HGMP)_2Mo_5O_{15}]$•$7H_2O$ (**1**).

*Flack H. On enantiomorph-polarity estimation. Acta Cryst A 1983, **39**(6): 876-881.

4. Supporting Illustrations

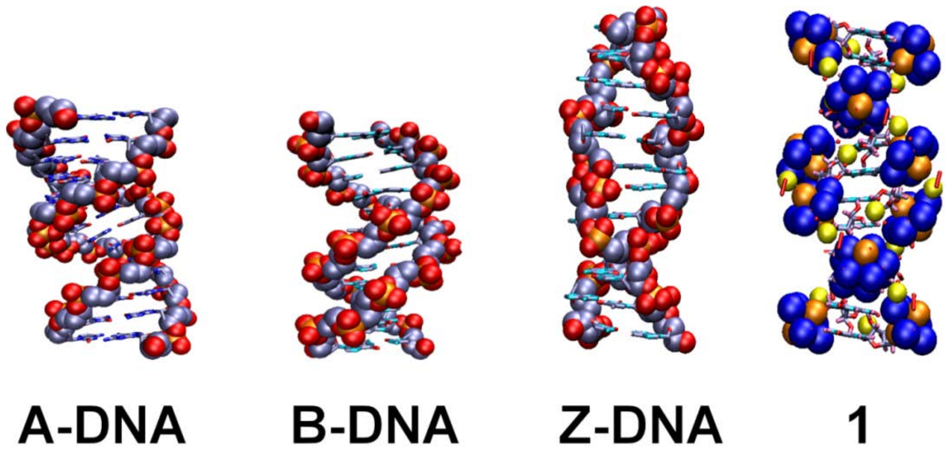

Figure S1: Images of A-, B- and Z-DNA as well as of compound **1**. The images were produced using VMD-software, using NDB-files pdb4okl (A-DNA), pdb4c64 (B-DNA), pdb1vty (Z-DNA). C ice blue, N light blue, O red, P orange, Mo dark blue. Hydrogen atoms omitted for clarity.[7]

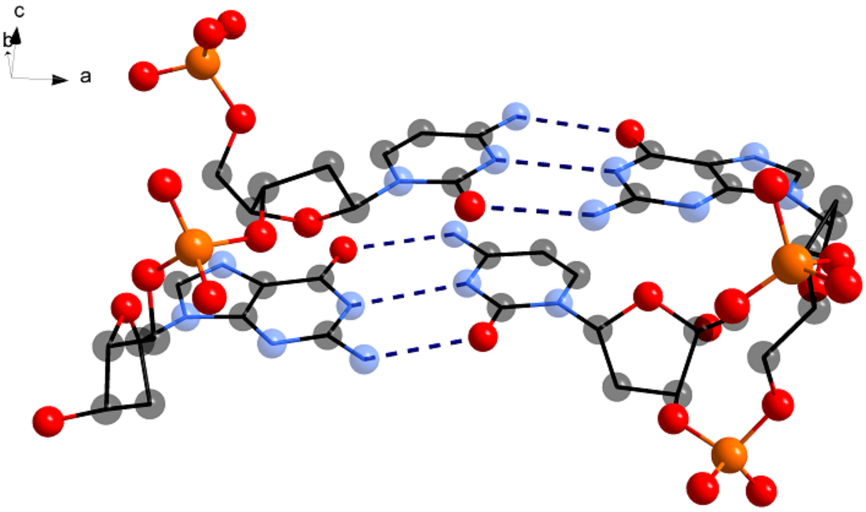

Figure S2. Dimer of Z-DNA.[8] Colour scheme: C black, N light blue, O red, P orange. Hydrogen atoms omitted for clarity. Same scheme applied in the subsequent figures.

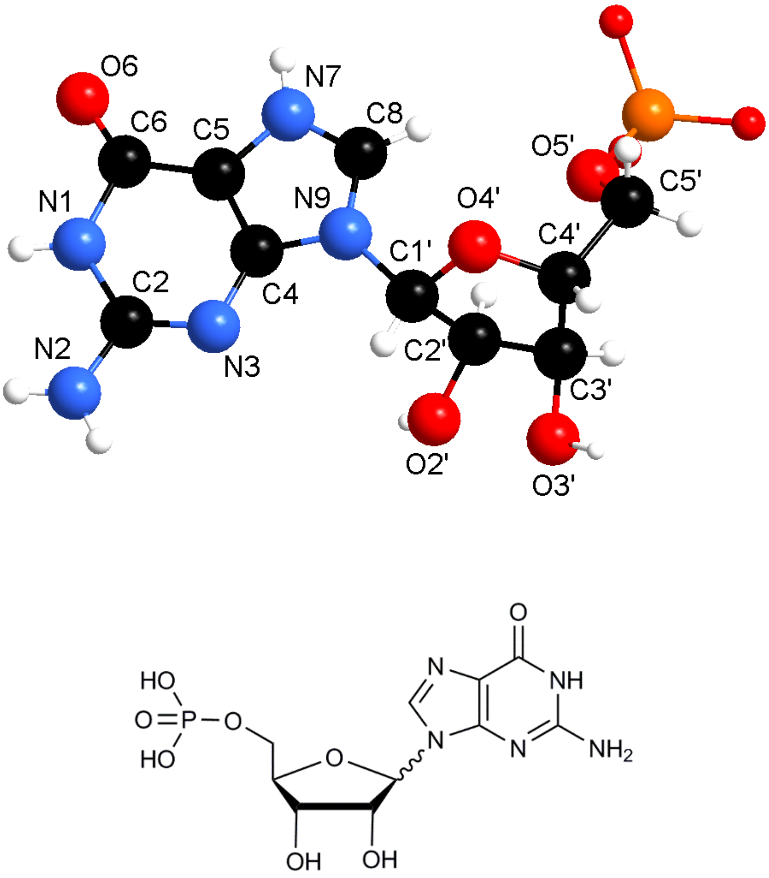

Figure S3. Atom numbering in guanosine monophosphate (**2**, GMP). H white. Specific stereochemistry of molecule also shown.

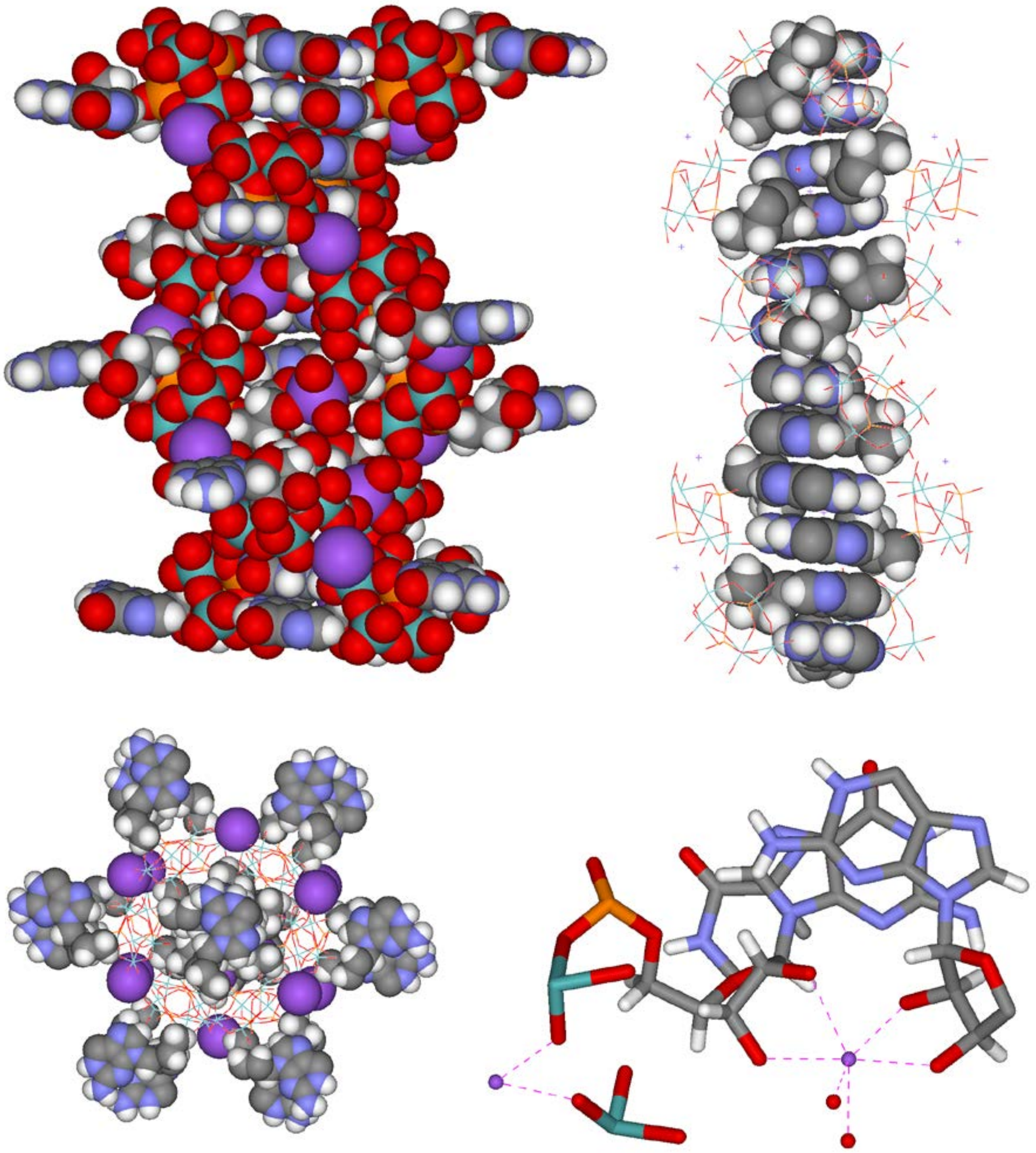

Figure S4. Top left: The helix from the side in CPK. Top right: The central core of the structure of **1**, in this case showing 12 guanosines pi-stacked (*ca.* 3.51 Å) giving a *ca.* 4 nm stack. Bottom left: A view down the Z axis of the helix. Bottomg right: The sodium cations coordinating to adjacent clusters can be seen (*ca.* 2.56 Å) as well as coordinating to two ribose units in a cis-chelate (*ca.* 2.41 Å). The clusters are shown in wire frame, the Guanosines in CPK (both images). Colour scheme: O – red; Na – purple; C – grey; N – blue; Mo – green; P – orange; H – white.

Table S2. A complete version of Table 1 showing the structural features of ideal B-, Z-DNA and $Na_2[(HGMP)_2Mo_5O_{15}]\cdot 7H_2O$ (**1**). *Guanine rings form H-bonded stacks as oppose to classical base pairing.

	**B-DNA[9]**	**Z-DNA[9]**	**Compound 1**
Helical Sense	Right Handed	Left Handed	Left Handed
Diameter	~20 Å	~18 Å	~32 Å
Base Pairs per Helical Turn	10	6 pairs	6 pairs*
Helical Twist per Base Pair	36°	60° per dimer	60° per dimer
Angle within Dimers		9°	4°
Rise per Turn	34 Å	45 Å	42 Å
Rise per Base Pair along the Central Axis	3.4 Å	7.4 Å per dimer	7.0 Å per dimer
Interplanar Ring Distance	3.4 Å	3.4 Å within dimers	3.4 Å within dimers
Base Normal to the Helix Axis	6°	7°	7°
Sugar Pucker	C2'-*endo*	C2'-*endo* for pyrimidines; C3'-*endo* for purines	C2'-*endo*
Glycosidic Bond	Anti	Anti for pyrimidines; syn for purines	Anti

5. NMR Spectroscopy

5.1 NMR Signals

^{1}H NMR (400 MHz, c = 28 mg/ml, pD = 2.60, ppm): 4.48 (m, 1H, $H^{4'}$), 4.53 (m, 1H, $H^{5'}$), 4.60 (m, 1H, $H^{5'}$), 4.72 (m, 1H, $H^{3'}$), 4.96 (m, 1H, $H^{2'}$), 6.00 (d, $^{3}J_{H1'H2'} \approx 6.3$ Hz, 1H, $H^{1'}$), 8.68 (s, 1H, H^{8}). ^{13}C NMR (101 MHz, c = 28 mg/ml, pD = 2.60, ppm): 68.0 (d, $^{2}J_{C5'P} \approx 4.4$ Hz, $C^{5'}$), 73.9 ($C^{3'}$), 77.0 ($C^{2'}$), 87.6 (d, $^{3}J_{C4'P} \approx 9.7$ Hz, C^{4R}), 90.3 ($C^{1'}$), 140.2 (C^{8}), 154.0, 157.3, 160.3 (C^{2}, C^{4}, C^{6}). ^{31}P NMR (162 MHz, c = 5.6 mg/ml, pD = 2.92, ppm): 0.97 (dd, $^{3}J_{PH5'} \approx 3.9$ Hz, $^{3}J_{PH5'} \approx 7.8$ Hz).

5.2 P^{31}-NMR Spectrum

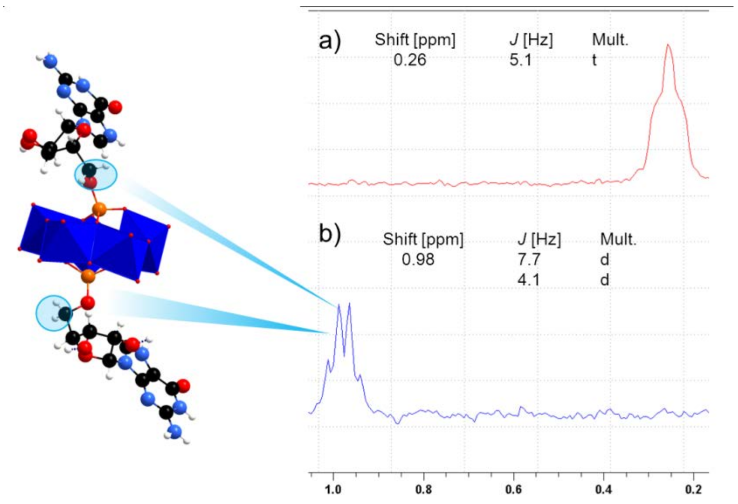

Figure S5. a) ^{31}P-NMR-Spectrum of GMP (c = 2.4 mg/ml; pD = 2.92); b) ^{31}P-NMR-Spectrum of compound **1** (c = 5.6 mg/ml ; pD = 2.92).The protons coupling to the P-nucleus are indicated by blue circles. Mo blue polyhedra.

5.3 Low field region of the ^{1}H-NMR Spectrum

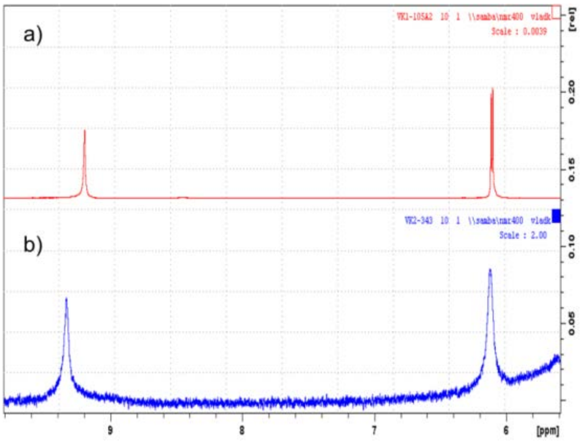

Figure S6. Low field region of the ^{1}H-NMR spectrum of **1**: a) c = 5.6 mg/ml; pD = 2.92; b) c = 35.9 mg/ml; pD = 2.15.

The ^{1}H-NMR spectra of **1** are harder to interpret than the ^{31}P-NMR ones, as protons of the ribose ring couple with one another, splitting the signals into broad multiplets. The only exception is H1' attached to the carbon bonded to the purine ring. Its coupling to H2' produces a well-defined doublet (Figure S5). The exact positions of the signals are dependent on the concentration and acidity of the NMR-samples due to agglomeration of guanine rings upon acidification.[10] Furthermore, the assembly of very large supramolecular aggregates leads to increased viscosity and eventual gelation of the samples.[11] In turn this phenomenon results in a decrease of the signal-to-noise ratio in ^{1}H NMR spectra and eventual disappearance of the peaks corresponding to assembled **1** (due to intensified spin-lattice relaxation), leaving only signals resulting from traces of unassembled **1** (compare Figure S5a and b).[12] As a result of these issues, detailed NMR studies, such as DOSY, of the assembly was not possible.

6. Ion mobility - Mass Spec

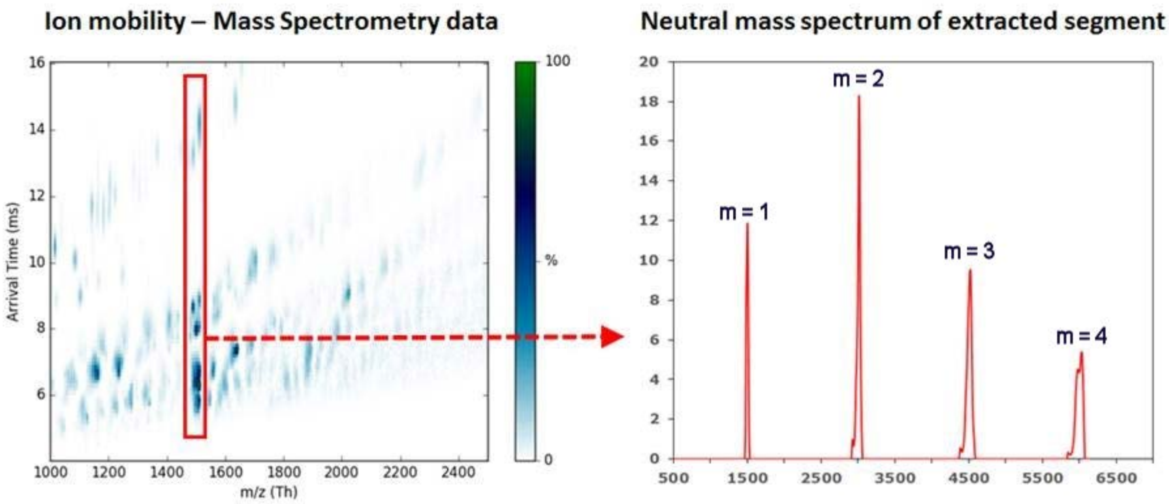

Figure S7. IMS-MS spectrum and interpretation as a neutral mass spectrum. This reveals a series of oligomeric peaks which may be assigned to a $[(C_{10}H_{13}N_5O_8P)_2(Mo_5O_{15})_1(K)W(Na)_X\ (H)_Y(H2O)_Z]_m$, suggeting the cluster to be intact in aqueous solution and demonstrating its propensity to aggregate.

7. Circular and Linear Dichroism

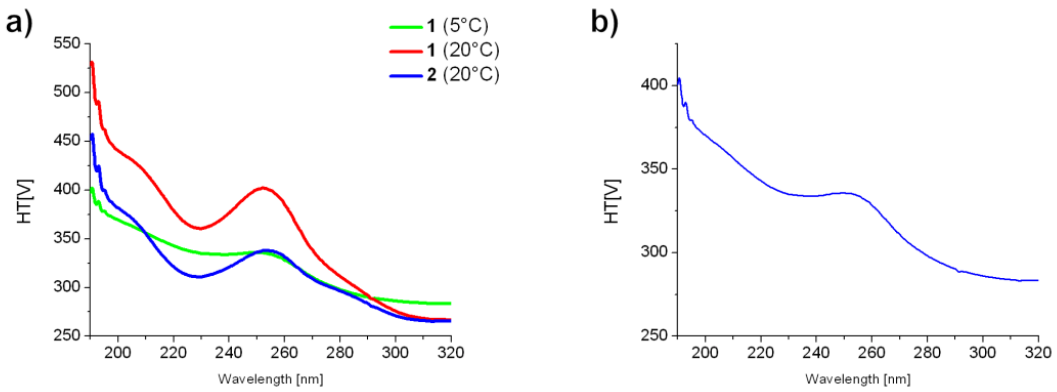

Figure S8. HT [V] spectra corresponding to the CD-spectra in figure **2**.

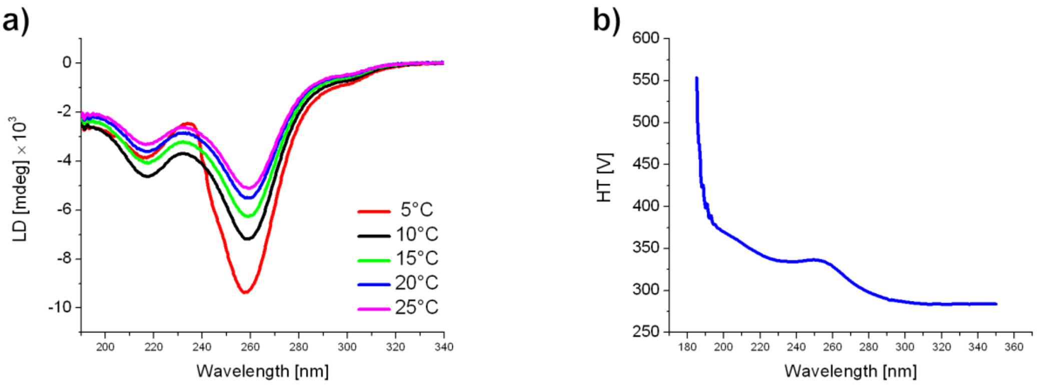

Figure S9. Temperature dependence of LD spectrum of **1** after cooling the solutions down to 5°C. All spectra were measured using a concentration of 4mM and pH 1.2. The sample was measured in a quartz demountable cell of pathlength ~ 0.001cm. The linear dichroism spectral intensity decreases with increasing temperature which suggests the sample is becoming less oriented (i. e. number of monomers organized in secondary structures decrease) at higher temperatures.

8. Atomic Force Microscopy

8.1 Three days solution ageing

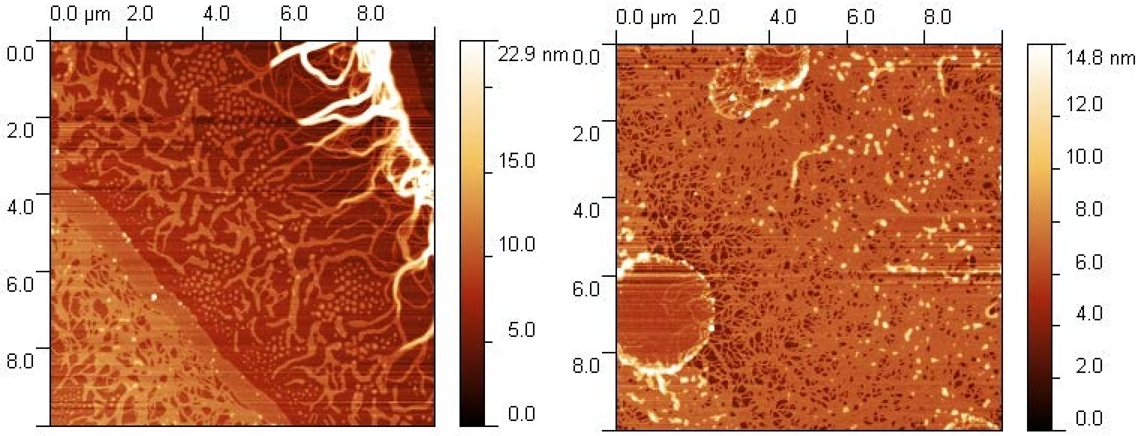

Figure S10: AFM height pictures illustrating the effects of local concentration on the formation of the fibers. These pictures have been taken after ageing a solution of **1** for 3 days, and drop cast 10 μl of it in a freshly cleaved mica surface. The pictures have been taken in the semi-contact mode.

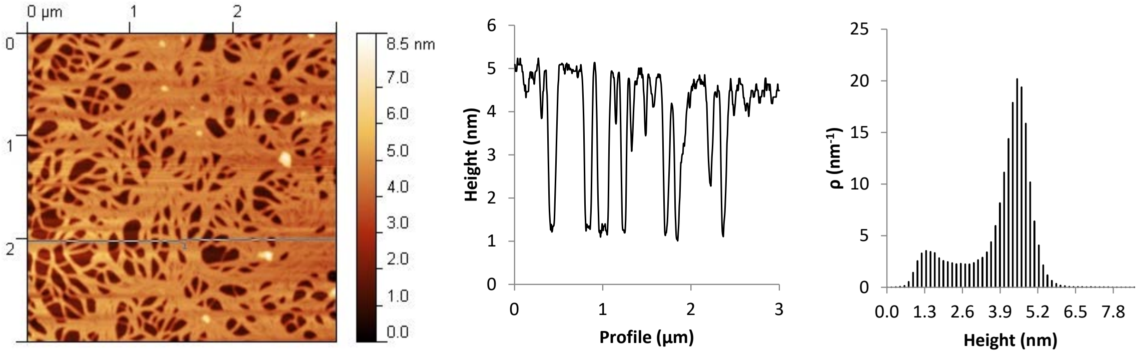

Figure S11: Left: AFM height picture, semi-contact mode; Middle: profile extract; Right: height distribution

8.2 Five days solution ageing

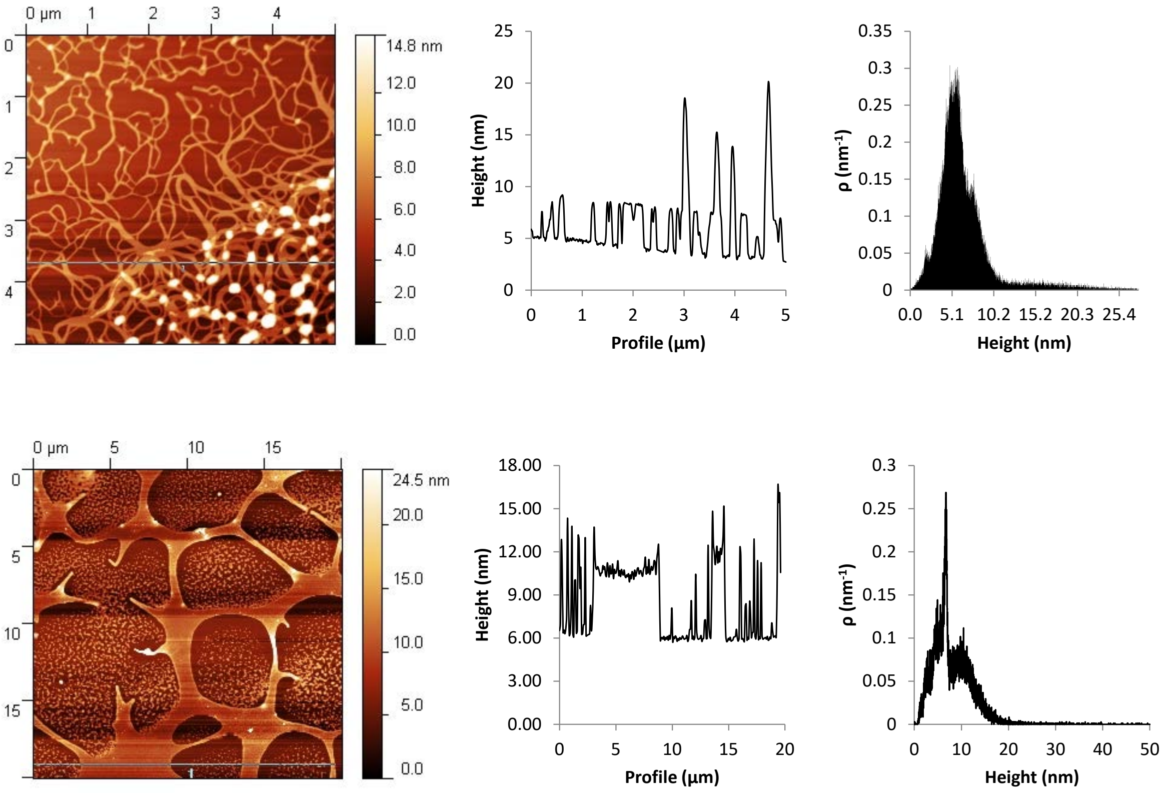

Figure S12: Two examples of fibers' formation seen in the semi-contact mode by AFM on a freshly cleaved mica surface. Left top and bottom: AFM Height pictures; Middle top and bottom: corresponding profile extracts; Right top and bottom: size distribution.

As previously reported in other hybrid systems,[13,14] the features seen on the surface by AFM depend highly on local concentration. In some areas a tight network of fibres is observed (see Figure S10) systematically highlighting a height of around 3.5 to 4.0

nm (Figure S11). This height is consistent with the fibres shown in all the AFM pictures displayed here (and discarding the few dots consisting of aggregated material). It also seems that ageing the solution doesn't have an important impact on the features seen on the surfaces. Guanosine self-assembly has already been studied by AFM and is known to form fibre-like structures on mica.[15-17] However in most reports the height of these fibres is between 1.5-2.0 nm. The difference with the 3.5-4.0 nm measured in this study is the consequence of the presence of the inorganic core, and is consistent with the helicoidal diameter measured on the crystal structure (3.85 nm). However, because the helical pitch is in the order of angstrom and because the diameter of the tip of the cantilever is 10 nm, it is not possible to see any helical structure on these pictures. Because the freshly cleaved mica surface is negatively charged, it is expected that the structure will not grow from a dimer of hybrids POM-guanosine, as this would require the anion to interact directly with the surface, but that the guanosine moiety not involved in the dimerization would interact with the surface. It also seems that the structures do not tend to pile up vertically but are aggregating along the surface, as highlighted by the pictures illustrating the effects of local concentration. The slight difference between the height of the fibres and the diameter of the helix could be explained by the guanosine interacting not only *via* its free amine group, but lying flat on the surface in order to maximize the stabilizing interaction, leading to a more compact structure.

9. DNA Annealing Experiments

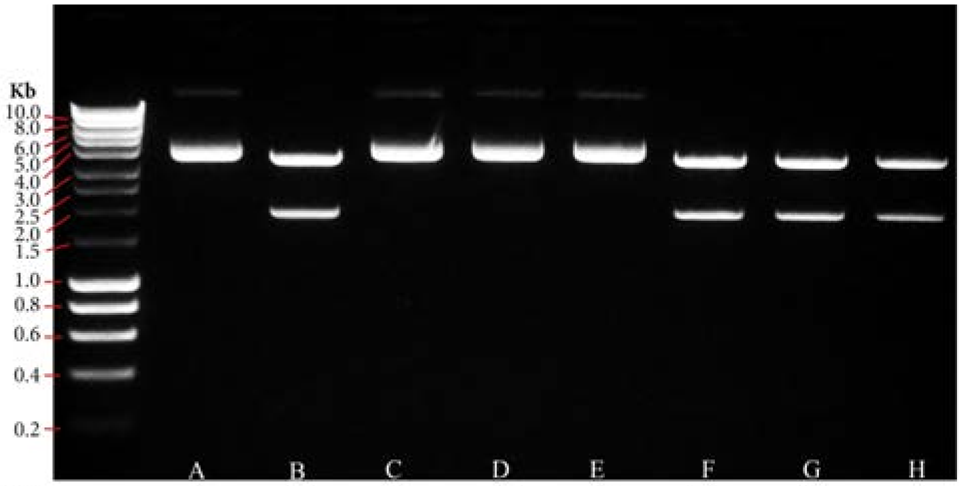

		A	B	C	D	E	F	G	H
ds-DNA	-	+	+	+	+	+	+	+	+
Enzyme	-	-	+	-	-	-	+	+	+
**1**	-	-	-	+	+	-	+	+	-
**5**	-	-	-	-	-	+	-	-	+
DNA Marker	+	-	-	-	-	-	-	-	-

Figure S13: Double stranded DNA was incubated with and without NDEI (restriction endonuclease) in the presence of compounds **1** and **5**. The results show no difference between the incubation only double stranded DNA and the same experiment in the presence of compounds **1** and **5**. In addition the experiment indicates that both compounds did not affect the activity of the restriction enzyme.

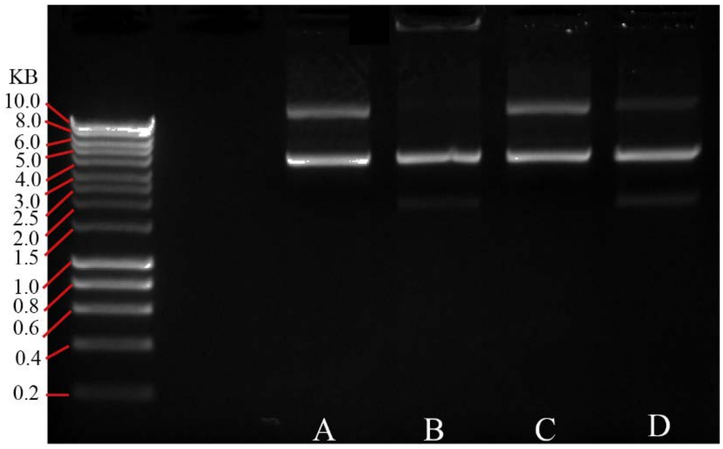

		A	B	C	D
ds-DNA	-	+	-	+	-
ss-DNA	-	-	+	-	+
GMP	-	+	+	-	-
DNA Marker	+	-	-	-	-

Figure S14: DNA interactions with GMP. ds-DNA = double stranded pGLO plasmid DNA, ss-DNA = single stranded pGLO plasmid DNA.

10. Differential Thermal Analysis / Thermogravimetry

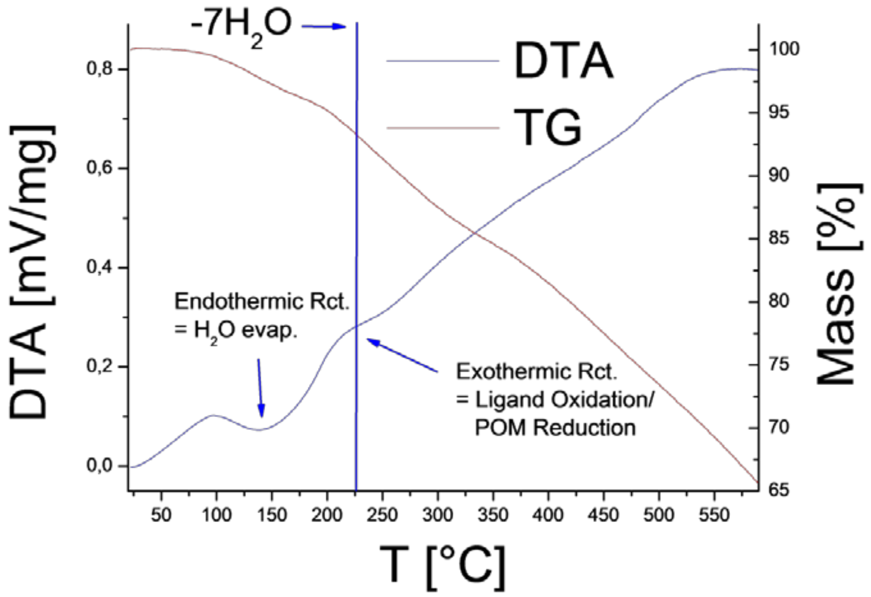

Figure S15: DTA/TG curve of the compound **1**.

11. Gel Electrophoresis

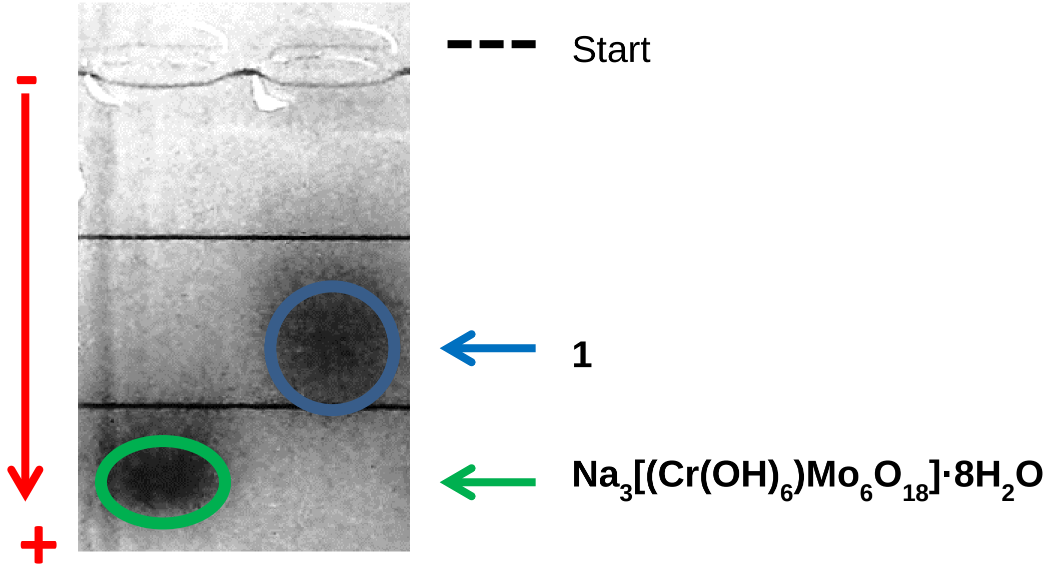

Figure S16: Gel electrophoresis of compound **1** against an external reference. The image was rendered in greyscale, and contrast adjusted to most clearly show resolution of the species. The compound $Na_3[(Cr(OH)_6)Mo_6O_{18}]\cdot 8H_2O$ was chosen as the reference polyoxometalate, because it is stable under the buffer conditions (pH =

4.6). The $[(Cr(OH)_6)Mo_6O_{18}]^{3-}$-anion displays higher charge and smaller size than the hybrid anion of the compound **1** and travelled accordingly faster. The single spot left by compound **1** upon staining is an indication that the hybrid POM is stable at quite low concentration under the buffer conditions.

The 1% agarose gel was prepared in an aqueous sodium acetate / acetic acid buffer (70 mM, pH = 4.6; agarose was purchased from Sigma Aldrich and used without further purification). The electrophoresis was performed for 10 min (U = 100 V). The solutions of both – the compound **1** and references were prepared at the concentration of 13 mg/ml in the same buffer. In order to visualise both polyoxometalates in the gel, they were reduced by reaction with $Na_2S_2O_4$ solution (1% in 100 ml H_2O acidified with 4 drops of HCl (conc.)) to produce blue reduced derivatives. The external reference compound $Na_3[(Cr(OH)_6)Mo_6O_{18}]\cdot 8H_2O$ was prepared according to the published procedure.[18]

12. List of Movies

Movie S1: The helix of the compound **1**.

Movie S2: Z-DNA.

13. References

1. Hill L. M. R., George G. N., Duhme-Klair A.-K., Young C. G. Solution structural studies of molybdate–nucleotide polyanions. *J. Inorg. Biochem.* **88,** 274-283 (2002).
2. Clark R. C., Reid J. S. The analytical calculation of absorption in multifaceted crystals. *Acta Crystallogr. A* **51,** 887-897 (1995).
3. Blessing R. An empirical correction for absorption anisotropy. *Acta Crystallogr. A* **51,** 33-38 (1995).
4. Sheldrick G. A short history of SHELX. *Acta Crystallogr. A* 2008, **64,** 112-122.
5. Farrugia L. WinGX suite for small-molecule single-crystal crystallography. *J. Appl. Crystallogr.* **32,** 837-838 (1999).
6. Sheldrick G. *Shelxl-2013.* (Göttingen, Germany, 2013).
7. Humphrey W., Dalke A., Schulten K. VMD: Visual molecular dynamics. *J. Mol. Graphics.* **14,** 33-38 (1996).

8. Drozdzal P., Gilski M., Kierzek R., Lomozik L., Jaskolski M. Ultrahigh-resolution crystal structures of Z-DNA in complex with Mn^{2+} and Zn^{2+} ions. *Acta Crystallogr. D* **69,** 1180-1190 (2013).

9. Wang, A. H. J., Quigley, G. J., Kolpak, F. J., Crawford, J. L., Boom, J. H. van, Marel, G. van der, Rich, A., Molecular structure of a left-handed double helical DNA fragment at atomic resolution. *Nature*, **282,** 680-686, (1979).

10. Wong A., Ida R., Spindler L., Wu G. Disodium guanosine 5'-monophosphate self-associates into nanoscale cylinders at pH 8: a combined diffusion NMR spectroscopy and dynamic light scattering study. *J. Am. Chem. Soc.* **127,** 6990-6998, (2005).

11. Foster J., Piepenbrock M.-O. M., Lloyd G., Clarke N., Howard J., Steed J. Anion-switchable supramolecular gels for controlling pharmaceutical crystal growth. *Nat. Chem.* **2,** 1037-1043 (2010).

12. Andrasko J. Water in agarose gels studied by nuclear magnetic resonance relaxation in the rotating frame. *Biophys. J.* **15,** 1235-1243, (1975).

13. Musumeci C., Luzio A., Pradeep C. P., Miras H. N., Rosnes M. H., Song Y.-F.*, et al.* Programmable Surface Architectures Derived from Hybrid Polyoxometalate-Based Clusters. *J. Phys. Chem. C* **115,** 4446-4455, (2011).

14. Hutin M., Yvon C., Yan J., Macdonell A., Long D.-L., Cronin L. Programming the assembly of carboxylic acid-functionalised hybrid polyoxometalates. *Cryst. Eng. Comm.* **15,** 4422-4430, (2013).

15. Kunstelj K., Federiconi F., Spindler L., Drevenšek-Olenik I. Self-organization of guanosine 5′-monophosphate on mica. *Colloids and Surfaces B: Biointerfaces* **59,** 120-127 (2007).

16. Oliveira Brett A. M., Chiorcea Paquim A. M., Diculescu V., Oretskaya T. S. Synthetic oligonucleotides: AFM characterisation and electroanalytical studies. *Bioelectrochemistry* **67,** 181-190, (2005).

17. Li Y., Dong M., Otzen D., Yao Y., Liu B., Besenbacher F., *et al.* Influence of tunable external stimuli on the self-assembly of guanosine supramolecular nanostructures studied by atomic force microscope. *Langmuir* **25,** 13432-13437, (2009).

18. Perloff A. Crystal structure of sodium hexamolybdochromate(III) octahydrate, $Na_3(CrMo_6O_{24}H_6).8H_2O$. *Inorg. Chem.* **9,** 2228-2239 (1970).

19. Marty M. T., Baldwin A. J., Marklund E. G., Hochberg G. K. a., Benesch J. L. Robinson C. V. *Anal. Chem.*, **87**, 4370-4376 (2015).
